# Germinal Center Alloantibody Responses Mediate Progression of Chronic Allograft Injury

**DOI:** 10.3389/fimmu.2018.03038

**Published:** 2019-01-23

**Authors:** Manu Chhabra, Jawaher Alsughayyir, M. Saeed Qureshi, Mekhola Mallik, Jason M. Ali, Ivonne Gamper, Ellen L. Moseley, Sarah Peacock, Vasilis Kosmoliaptsis, Martin J. Goddard, Michelle A. Linterman, Reza Motallebzadeh, Gavin J. Pettigrew

**Affiliations:** ^1^School of Clinical Medicine, University of Cambridge, Cambridge, United Kingdom; ^2^Department of Pathology, Papworth Hospital, Papworth Everard, United Kingdom; ^3^Histocompatibility and Immunogenetics Laboratory, Cambridge University Hospitals NHS Foundation Trust, Cambridge, United Kingdom; ^4^Laboratory of Lymphocyte Signalling and Development, Babraham Institute, Cambridge, United Kingdom; ^5^Division of Surgery and Interventional Science, University College London, London, United Kingdom; ^6^Centre for Transplantation, Department of Renal Medicine, University College London, London, United Kingdom; ^7^Institute of Immunity and Transplantation, University College London, London, United Kingdom

**Keywords:** allograft, humoral allograft rejection, germinal center (GC), extrafollicular B cell response, transplantation

## Abstract

Different profiles of alloantibody responses are observed in the clinic, with those that persist, often despite targeted treatment, associated with poorer long-term transplant outcomes. Although such responses would suggest an underlying germinal center (GC) response, the relationship to cellular events within the allospecific B cell population is unclear. Here we examine the contribution of germinal center (GC) humoral alloimmunity to chronic antibody mediated rejection (AMR). A murine model of chronic AMR was developed in which T cell deficient (*Tcrbd*^−/−^) C57BL/6 recipients were challenged with MHC-mismatched BALB/c heart allografts and T cell help provided by reconstituting with 10^3^ “TCR75” CD4 T cells that recognize self-restricted allopeptide derived from the H-2K^d^ MHC class I alloantigen. Reconstituted recipients developed Ig-switched anti-K^d^ alloantibody responses that were slow to develop, but long-lived, with confocal immunofluorescence and flow cytometric characterization of responding H-2K^d^-allospecific B cells confirming persistent splenic GC activity. This was associated with T follicular helper (T_FH_) cell differentiation of the transferred TCR75 CD4 T cells. Heart grafts developed progressive allograft vasculopathy, and were rejected chronically (MST 50 days), with explanted allografts displaying features of humoral vascular rejection. Critically, late alloantibody responses were abolished, and heart grafts survived indefinitely, in recipients reconstituted with *Sh2d1a*^−/−^ TCR75 CD4 T cells that were genetically incapable of providing T_FH_ cell function. The GC response was associated with affinity maturation of the anti-K^d^ alloantibody response, and its contribution to progression of allograft vasculopathy related principally to secretion of alloantibody, rather than to enhanced alloreactive T cell priming, because grafts survived long-term when B cells could present alloantigen, but not secrete alloantibody. Similarly, sera sampled at late time points from chronically-rejecting recipients induced more vigorous donor endothelial responses *in vitro* than sera sampled earlier after transplantation. In summary, our results suggest that chronic AMR and progression of allograft vasculopathy is dependent upon allospecific GC activity, with critical help provided by T_FH_ cells. Clinical strategies that target the T_FH_ cell subset may hold therapeutic potential.

This work is composed of two parts, of which this is Part II. Please read also Part I: Alsughayyir et al., 2019.

## Introduction

The contribution of humoral alloimmunity to transplant failure is increasingly recognized (1–3). In addition to acute antibody mediated rejection (AMR) that typically occurs early after transplantation [considered in the companion paper (4)], donor specific alloantibody (DSA) responses are associated with chronic graft dysfunction, progressive allograft vasculopathy and early graft failure (5). Chronic AMR is less well-defined, and not recognized as a distinct clinical entity for some organs (6). Nevertheless, the development of DSA against mismatched MHC antigen is now known to be a major risk factor for early transplant failure of liver (7, 8) kidney (9–12), lung (13, 14), and heart (15, 16) allografts. Chronic AMR is most clearly described following kidney transplantation and presents as transplant glomerulopathy (17–19), with an associated graft loss of 50% at 18 months (10).

The hallmark feature of chronic AMR—progressive allograft vasculopathy—is thought to be a culmination of cycles of insidious injury and repair, and it is notable that the associated alloantibody response usually persists long after its first detection (20, 21). Such long-lived antibody responses are typically mediated by long-lived plasma cells (LLPCs) that are deposited in the bone marrow (22), and that, in turn, are generally considered a product of an affinity-matured germinal center reaction (GC) (23–25). However, somatic hypermutation has been described within extrafollicular foci (26, 27), and, notably, the allospecific GC response has not yet been delineated, because of the difficulty in accessing tissue in human transplant patients, and because murine models of chronic AMR are limited (28, 29). Thus, it is not yet known whether a GC response against MHC alloantigen is essential for the production of long-lived alloantibody that underpins the development of chronic AMR and allograft vasculopathy. Experimental confirmation of a central role for the GC response in chronic rejection would have immediate relevance for clinical transplantation, because the GC reaction is now known to require help for its initiation and maintenance from a specialized subset of PD-1^hi^CXCR5^hi^ CD4 T follicular helper (T_FH_) cells (30–33).

Here, we develop a murine cardiac model of chronic AMR to demonstrate that progression of allograft vasculopathy is dependent upon germinal center alloantibody responses that are associated with affinity maturation, and that are critically dependent upon the provision of help from T_FH_ cells that recognize processed target alloantigen via the indirect pathway.

## Materials and Methods

### Animals

C57BL/6 (BL/6; H-2^b^) and BALB/c mice (H-2^d^) were purchased from Charles River Laboratories (Margate, UK) and maintained according to the institutional guidelines of The University of Cambridge. T cell receptor-deficient mice (H-2^b^, *Tcrbd*^−/−^) BL/6.129P2-*Tcrb*^*tm*1*Mom*^*Tcrd*^*tm*1*Mom*^/J were purchased from the Jackson Laboratory (Bar Harbor, ME). C57BL/6 *Rag2*^−/−^ mice (H-2b) were gifted by Prof T. Rabbitts (Laboratory of Molecular Biology, Cambridge, UK). TCR-transgenic *Rag1*^−/−^ TCR75 mice (H-2^b^), specific for I-A^b^-restricted H-2K${}_{54--68}^{\text{d}}$ peptide (34) and C57BL/6-Tg(K^d^)RPb (BL/6.K^d^) mice, which express the full sequence of H-2K^d^ (35), were gifted by Prof. P. Bucy (University of Alabama, Birmingham, AL). BCR-transgenic SW_HEL_ (VH10${}_{\text{tar}}^{+/-}$ x LC2) mice (H-2^b^) specific for Hen Egg Lysozyme (HEL) protein (36) and BL/6.mHEL mice (H-2^b^, KLK3 Tg) that express membrane bound HEL (37) under the H-2K^b^ promoter, were gifted by Prof R. Brink (Garvan Institute of Medical Research, Darlinghusrt, Australia). BL/6 *Rag2*^−/−^ SW_HEL_ were generated by crossing SW_HEL_ BCR–transgenic mice crossed onto the *Rag2*^−/−^ background (38). *Sh2d1a*^−/−^ (SLAM-associated protein [SAP]-deficient) mice (39) were gifted by Dr S. Crotty (University of California, La Jolla, California). BL/6.K^d^ mice were crossed with BL/6.mHEL mice to create a BL/6 mHEL-K^d^ donor strain. Mice were bred and maintained in specific pathogen-free animal facilities and were maintained in individual ventilated cages in specific-pathogen free facilities and fed standard rodent feeds. Mice weighed between 18 and 22 g at the time of their use for *in vitro* experiments and transplants.

### Skin and Heterotopic Cardiac Transplantation

Full-thickness tail skin was sutured as 1 cm^2^ grafts onto the recipients' back. Vascularized cardiac allografts were transplanted intra-abdominally as previously described (40, 41). See also our companion paper (4).

### Histopathology

Heart graft rejection was defined as cessation of palpable myocardial contraction, confirmed at the time of explant. Grafts were excised at predetermined time points after transplantation and stored at −80°C or fixed in 10% buffered formalin. Cardiac allograft vasculopathy was assessed on elastin van Gieson-stained paraffin sections by morphometric analysis as previously described (42). All elastin-positive vessels in each section were evaluated, with approximately 10 vessels/heart analyzed. The severity of parenchymal allograft damage was scored on hematoxylin and eosin (H&E) stained paraffin sections by a cardiac histopathologist (EM and MG), blinded to the study groups, using a scale modified from the International Society for Heart and Lung transplantation (43) as follows: 0, no parenchymal damage; 1, < 30% parenchymal damage; 2, 30–60% parenchymal damage; 3, >60% parenchymal damage.

### Assay of Anti-H-2K^d^ Humoral Immunity

See our companion paper (4).

### Immunohistology and Confocal Imaging

Seven micrometer spleen and heart cryostat sections were air-dried and fixed in acetone. Primary mAbs specific for the following mouse epitopes were used for immunohistochemical/fluorescent staining: C4d (clone 16-D2 Abcam, Cambridge, UK), NK1.1 (PK136, Abcam) CD68 (ER-HR3, Abcam), mucosal addressin cell adhesion molecule (MAdCAM-1; clone MECA-367, Abcam), CD31 (Novus Biologicals, CO, USA), α-smooth muscle Actin (Thermo Fisher Scientific), and IgG-FITC (BD Biosciences, San Diego, CA, USA). Splenic GCs were identified by double-labeling sections with rat anti-mouse B220-APC (clone RA3-6B2) and rat anti mouse GL7-FITC (both BD Biosciences). Numbers of GL7^+^ GCs were expressed as a percentage of total B220^+^ lymphoid follicles (44). CD4 T cells within GCs were located with rat anti-mouse CD4-biotin (BD Biosciences) & Streptavidin-Alexa Fluor 555 (Thermo Fisher Scientific). Confocal images were captured with a Leica SP5 confocal microscope using LAS AF software, version 2.7.2.9586 (Leica Microsystems, Wetzlar, Germany).

### Alloantibody Purification From Serum Samples

IgG antibodies were purified from mouse serum samples using the Thermo Scientific Antibody Purification Kit (Thermo Fisher Scientific). Protein G spin columns were loaded with serum samples and binding buffer (0.1 M phosphate, 0.15 M sodium chloride; pH 7.2), centrifuged at 5,000 g and samples were eluted after addition of neutralization buffer followed by IgG elution buffer. A NanoDrop Microvolume Spectrophotometer was used to determine total IgG antibody concentrations using absorbance values at 280 nm. Samples were subsequently used in analysis of endothelial intracellular signaling.

### Endothelial Cell Migration Assay

*In vitro* wound-healing assay was performed as previously described (45). For endothelial cell culture, 10–14 day old neonatal hearts were digested with collagenase and endothelial cells labeled with biotin-conjugated antibodies against CD31 (clone MEC 13.3, BD Pharmingen), CD105 (clone MJ7/18, BioLegend, San Diego, CA, USA), and Isolectin B4 (clone B-1205, Vector, Burlingame, CA), and then separated using anti-biotin MicroBeads (Mitenyi Biotec) with an AutoMACS™ Separator (Mitenyi Biotec). Endothelial cells were cultured until 80–90% confluent and cells were subsequently incubated with medium lacking growth factors for 24 h to minimize baseline proliferation. A linear lesion was made in the cell monolayer across the diameter of the dish using a sterile 200 μl pipette tip. Cells were incubated with test sera (purified IgG) or control antibody for a further 24–36 h, fixed with paraformaldehyde (BD Cytofix kit, BD Biosciences), and then stained with 0.05% Crystal Violet solution. For each plate, six high power fields along the lesion were analyzed and degree of migration induced by test sera was calculated by determining the proportion of outward migration area in relation to a positive control [pooled hyperimmune anti-H-2K^d^ IgG serum recovered from BL/6 recipients of BALB/c skin grafts (SG)], normalized to 100%.

### Analysis of Endothelial Intracellular Signaling by Preparation of Cell Lysates and Western Blot

The ability of column-purified test sera from transplanted mice to induce phosphatidylinositol-3-kinase (PI3K)/Akt signaling of cultured BALB/c endothelial cells was tested as previously described (46) with minor modifications. For endothelial cell culture, 10–14 day old neonatal hearts were digested with collagenase and endothelial cells labeled with biotin-conjugated antibodies against CD31 (clone MEC 13.3, BD Biosciences), CD105 (clone MJ7/18, BioLegend), and Isolectin B4 (clone B-1205, Vector, Burlingame, CA), and then separated using anti-biotin MicroBeads (Mitenyi Biotec) with an AutoMACS^TM^ Separator (Mitenyi Biotec). Endothelial cells were cultured until 80–90% confluent and cells were subsequently incubated with medium lacking growth factors for 24 h to minimize baseline proliferation. Cultured BALB/c endothelial cells that were starved for 24 h in medium containing fetal calf serum without additional growth factors, were subsequently incubated with column-purified serum (see above) of interest (diluted to 1:100) for 30 min at 37°C. The supernatant was removed, cells were then washed and subsequently lysed with CelLytic M reagent (Sigma-Aldrich) containing protease inhibitor cocktail (Roche Life Sciences, Penzberg, Germany). Cell lysate was heated in sodium dodecyl sulfate lysis buffer, electrophoresed on 20% polyacrylamide gel (with approximately 10 μg of cell lysate per lane quantified using the protein quantification kit-rapid [Sigma-Aldrich]), and transferred to a polyvinylidene difluoride membrane. The membrane was blocked in 5% milk in phosphate-buffered saline (PBS) containing 0.1% Tween-20 for 1 h at room temperature, and incubated overnight at 4°C with phosphorylated-Akt (Ser473) mouse mAb (Cell Signaling Technology, Danvers, MA, USA) diluted to 1:1000 in blocking buffer. The blot was washed with PBS containing 2.5% Tween-20 followed by incubation in polyclonal HRP-conjugated rabbit anti-mouse IgG (Abcam) and then developed with chemiluminescence using standard procedure. Anti-GADPH (Abcam) was blotted as a loading control. Data depicted are representative of three independent experiments.

### Bio-Layer Interferometry (BLI)

Off-rate screening was performed on RED96 platform instrument ForteBio (Menlo Park, CA, USA). All experiments included biosensor pre-equilibration step in 1 × PBS buffer containing 0.1% BSA, 0.02% Tween-20, and 0.02% NaN_3_ (pH 7.4); hereafter referred to as kinetics buffer for 10 min, and data were collected at 27°C with agitation at 1,000 rpm. Biotinylated MHC class I K^d^ antigen (1.25 μg/mL) was captured on streptavidin coated biosensors (ForteBio) at a maximal threshold of 1.5 nm in kinetics buffer. After MHC antigen capture, sensors were blocked for 60 s with naive serum obtained from BL/6 *Tcrbd*^−/−^ mice. After blocking, sensors were transferred to corresponding wells containing immune serum diluted in kinetics buffer for association over 1,000 s, followed by transfer to dissociation wells containing diluted naïve serum over a period of 1,000 s. Each biological sample was tested at two dilutions: 1:20 and 1:40. Dissociation rate constants for each sample were calculated by applying a 1:1 interaction model (fitting local, partial), and double referenced with both reference biosensor (no biotinylated MHC class I K^d^ antigen loading) and sample reference (naïve BL/6 serum). Control experiments were performed using non-relevant control anti-HEL IgG antibody (obtained from sera of BL/6 SW_HEL_ recipients challenged with hearts from BL/6.mHEL mice) and showed no binding to sensors loaded with biotinylated K^d^ antigen. Data analysis was performed using the ForteBio data analysis software 7.0.1.5.

### Statistical Analysis

Data is presented as mean ± S.E.M where appropriate, with each animal constituting one biological replicate where indicated. Unpaired *t-*tests and Mann-Whitney *U*-tests were used for analysis of parametric data and non-parametric data, respectively. Two-way ANOVA was employed for comparison of anti-H-2K^d^ IgG alloantibody responses. Graft survival is depicted using Kaplan-Meier analysis and groups compared by log-rank (Mantel-Cox) testing. Analysis was conducted using GraphPad 4 (Graph- Pad Software, San Diego, CA, USA). Values of *P* < 0.05 were considered significant.

### Study Approval

This research has been regulated under the Animals (Scientific Procedures) Act 1986 Amendment Regulations 2012 following ethical review by the University of Cambridge Animal Welfare and Ethical Review Body (AWERB). All surgery was performed under inhalational anesthesia and every effort was made to minimize suffering. Human transmyocardial biopsy samples were obtained through the Papworth Hospital Research Tissue Bank (REC Ref: 08/H0304/56+5), project number T02333: “Visualization of tissue components in cardiac tissue post-transplantation.” All subjects gave written informed consent in accordance with the Declaration of Helsinki.

## Results

### Development of Model of Chronic Alloantibody Mediated Allograft Rejection

**Figure 1:**
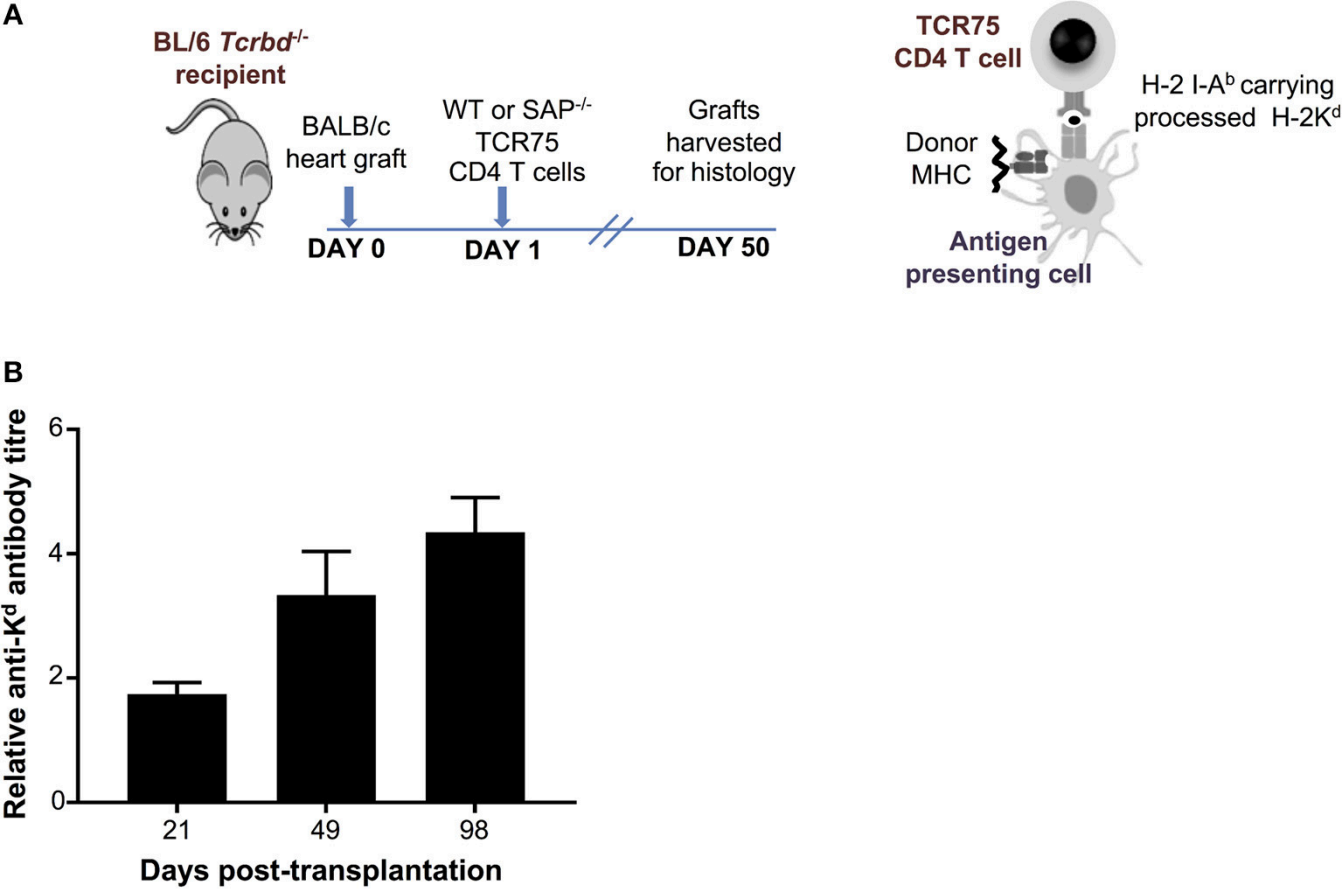
Development and characterization of murine model of antibody-mediated heart allograft rejection. **(A)** Figure is adapted from our companion paper (4). BL/6 *Tcrbd*^−/−^ recipients of BALB/c heart allografts were either unmodified (no cells group) or reconstituted the day after with either wild-type or SAP-deficient CD4 T cells from TCR transgenic *Rag1*^−/−^ TCR75 animals that recognize I-A^b^-restricted H-2K${}_{54-68}^{\text{d}}$ peptide. **(B)** ELISA assays of serum samples obtained from BL/6 *Tcrbd*^−/−^ recipients of BALB/c hearts and reconstituted with 10^3^ TCR75 CD4 T cells (*n* = 4) shows progressive increase of anti-H-2K^d^ IgG alloantibody until day 100.

As detailed in the companion paper (4), we developed a murine model of cardiac allograft rejection in which the humoral alloimmune response was likely the sole effector mechanism of graft rejection, because the recipients otherwise lacked CD8 T cells or direct-pathway CD4 T cells with the potential for generating cellular cytotoxicity directed against the graft. Thus, T cell deficient *Tcrbd*^−/−^ C57BL/6 recipients of a BALB/c heart allograft were reconstituted the day after transplant with TCR75 CD4 T cells (34, 35) that recognize donor MHC class I H-2K^d^ alloantigen as processed allopeptide via the indirect pathway [Figure 1A; see also companion paper (4)]. Recipient mice reconstituted with as few as 10^3^ TCR75 CD4 T cells generated long-lasting anti-H-2K^d^ IgG alloantibody, that was slow to develop, but was still evident 15 weeks after transplant (Figure 1B). This alloantibody response was associated with gradual failure of the BALB/c heart allograft [median survival time [MST]−50 days (4)], whereas BALB/c heart allografts survived indefinitely when transplanted into unmodified *Tcrbd*^−/−^ recipients.

**Figure 2:**
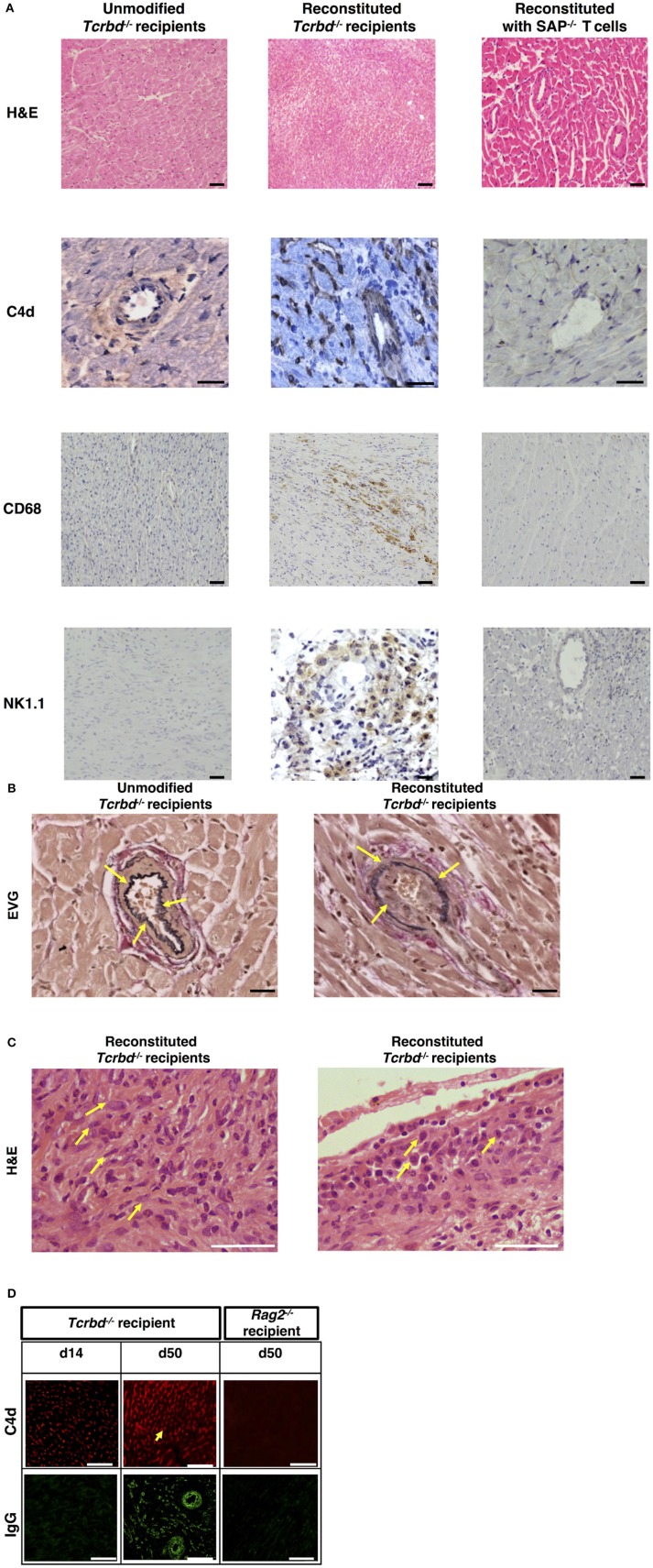
Histopathological confirmation of humoral rejection. **(A)** Representative photomicrographs of BALB/c hearts explanted at d50 from BL/6 *Tcrbd*^−/−^ recipients (reconstituted with 10^3^ wild-type [WT] TCR75 CD4 T cells) demonstrate: inflammatory infiltrates with myocyte loss and replacement fibrosis (on H&E staining); endothelial complement C4d deposition and areas of CD68^+^ (macrophage) and NK1.1^+^ (NK cell) staining, compared with grossly normal histology and negative staining in equivalent grafts from unmodified (not reconstituted) BL/6 *Tcrbd*^−/−^ recipients and BL/6 *Tcrbd*^−/−^ recipients reconstituted with *Sh2d1a*^−/−^ (SAP^−/−^) TCR75 CD4 T cells. Representative photomicrographs of **(B)** elastin van Gieson stained sections of d50 allografts from the wild-type reconstituted group depicting fibroproliferative arterial intimal thickening, compared to control unmodified *Tcrbd*^−/−^ recipients, and **(C)** H&E stained sections demonstrating inflammatory infiltrates consisting predominantly of (left) polymorphs and (right) occasional plasma cells (arrows). **(D)** Representative photomicrographs of immunofluorescence staining showing interstitial capillary/vascular staining (arrowed) for C4d (red) and IgG deposition (green) in BALB/c cardiac allografts explanted at days 14 and 50 from BL/6 *Tcrbd*^−/−^ recipients reconstituted with 10^3^ WT TCR75 CD4 T cells, and at day 50 from WT reconstituted *Rag2*^−/−^ recipients. Intensity of C4d and IgG staining increased from day 14 to day 50 in *Tcrbd*^−/−^ recipients reconstituted with WT TCR75 CD4 T cells. All images are representative of at least 5 animals: scale bars–A: 50 μm (H&E, CD68, and NK1.1 images, except for the NK1.1 image of the reconstituted *BL/6 Tcrbd*^−/−^ group which is at 75 μm) or 100 μm (C4d images), B: 100 μm, C: 150 μm, and D: top row; scale bar-−50 μm; bottom row; scale bar−100 μm.

Explanted heart allografts were assessed for histopathological features of AMR. Allografts, explanted at day 50 from the T cell reconstituted recipients, revealed chronic parenchymal injury, with diffuse myocyte loss and replacement fibrosis (Figure 2A). Areas of damage were associated with concentric proliferative vascular lesions, in keeping with a vasculopathic process causing chronic ischaemic injury (Figure 2B, *right*). These vascular lesions comprised endothelial and smooth muscle components, and were confined mainly to small intra-myocardial arteries and small/medium veins. Heart allografts transplanted into unmodified *Tcrbd*^−/−^ recipients remained disease-free (Figures 2A,B, left), confirming that allograft vasculopathy in the reconstituted recipients was mediated by the transferred TCR75 CD4 T cells. The histological features were not suggestive of chronic cellular rejection, in that although inflammatory infiltrates were present within the allografts, these consisted primarily of neutrophils and macrophages, with some plasma cells (Figure 2C); lymphocyte mediated myocyte damage-analogous to the pattern of cellular rejection observed in human heart allografts [“grade 2R” of the ISHLT grading system 43]–was not observed. Instead, a humoral effector component is strongly supported by: strong endothelial staining for C4d complement by-product associated with widespread IgG deposition (Figure 2D); the presence of macrosialin^pos^ (CD68) macrophages within the heart allograft; and by perivascular accumulations of NK cells (Figure 2A).

### Chronic Graft Injury Is Dependent Upon Germinal Center Alloantibody Responses

**Figure 3:**
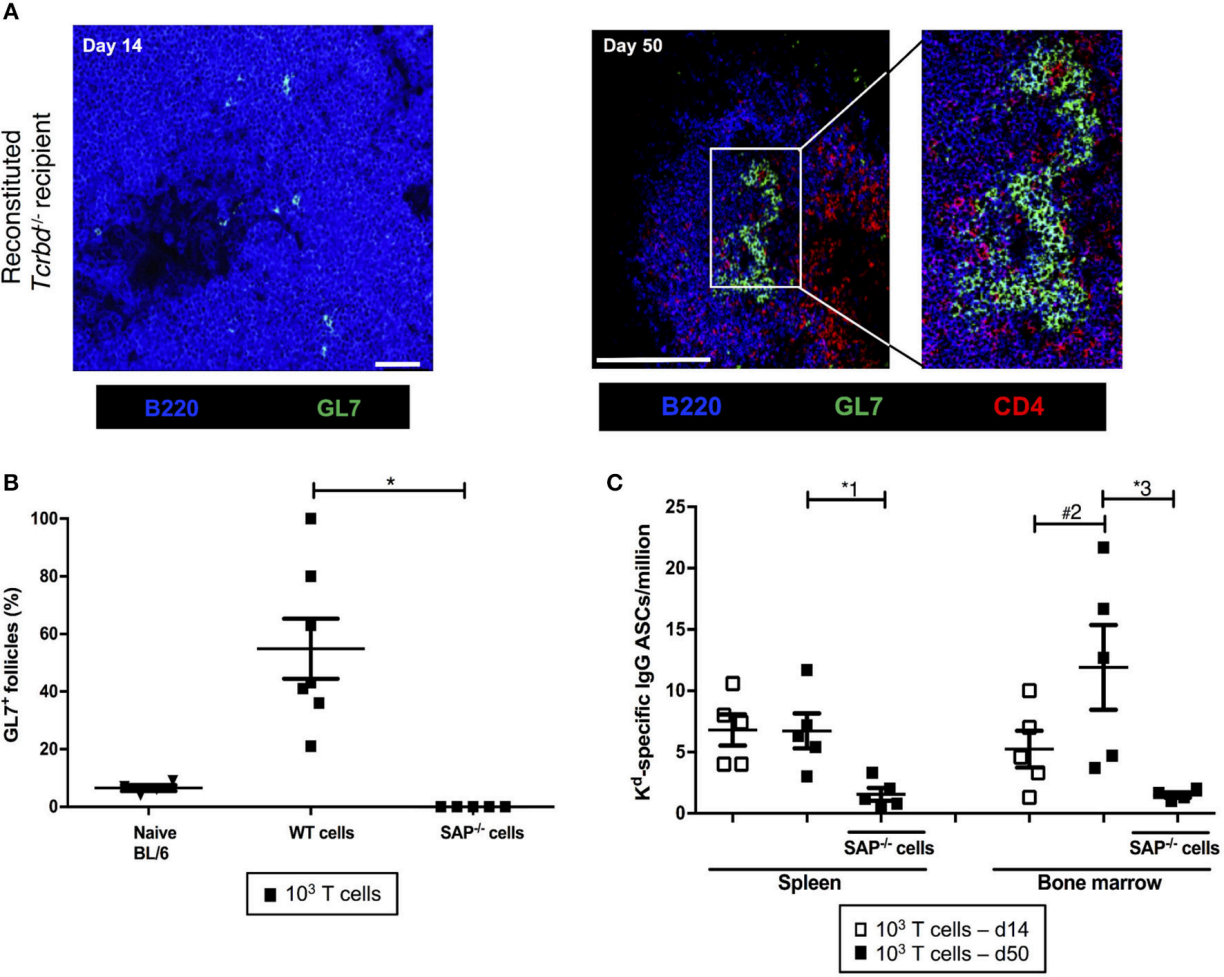
Germinal center responses and T_FH_ cell profile of adoptively transferred CD4 T cells following challenge a BALB/c heart allograft**. (A)** Representative confocal immunofluorescence photomicrograph of a splenic B cell follicle at d14 (left; scale bar−50 μm) and d50 (*right*; scale bar−250 μm) from reconstituted BL/6 *Tcrbd*^−/−^ recipients of BALB/c heart grafts stained with antibodies against B220 (B cells, blue), GL7 (GC B cells, green), and CD4 (T cells, red), demonstrating poorly defined GCs at d14 but by d50 a typical secondary follicle, with T_FH_ cells present within the GC *(inset)*, readily evident. **(B)** Histogram of secondary follicles expressed as percentage of total follicles within d50 spleens of BL/6 *Tcrbd*^−/−^ recipients either non-reconstituted (naïve) or adoptively transferred with 1 × 10^3^ WT or *Sh2d1a*^−/−^ (SAP^−/−^) TCR75 CD4 T cells. ^*^P = 0.01, Mann-Whitney test. **(C)** ELISPOT assay of splenic and bone-marrow anti-K^d^ IgG antibody secreting cells (ASCs) 50 days after transplantation. Numbers of bone marrow ASCs in recipients reconstituted with SAP^−/−^ TCR75 CD4 T cells were not above background. Data represents mean ± S.E.M of a minimum of 5 animals/group, with each dot representing the biological replicate in a distinct animal; ^*^^1^*P* = 0.02, ^#2^*P* = 0.15, ^*^^3^*P* = 0.02, Mann–Whitney test.

We sought to confirm whether the long-lasting humoral alloimmunity that underpinned progression of chronic allograft injury was the consequence of a GC response. In this regard, splenic GCs were poorly defined, rudimentary structures at 2 weeks (Figure 3A, left), but distinctly present 7 weeks after transplantation in the T cell-reconstituted recipient group (Figure 3A, right), with approximately half the B cell follicles staining for markers of GC differentiation (Figure 3B). In accordance, bone marrow deposition of H-2K^d^-specific plasma cells-an indicator of GC activity–was readily detectable by day 50 after transplantation, and had increased from day 14 (Figure 3C).

**Figure 4:**
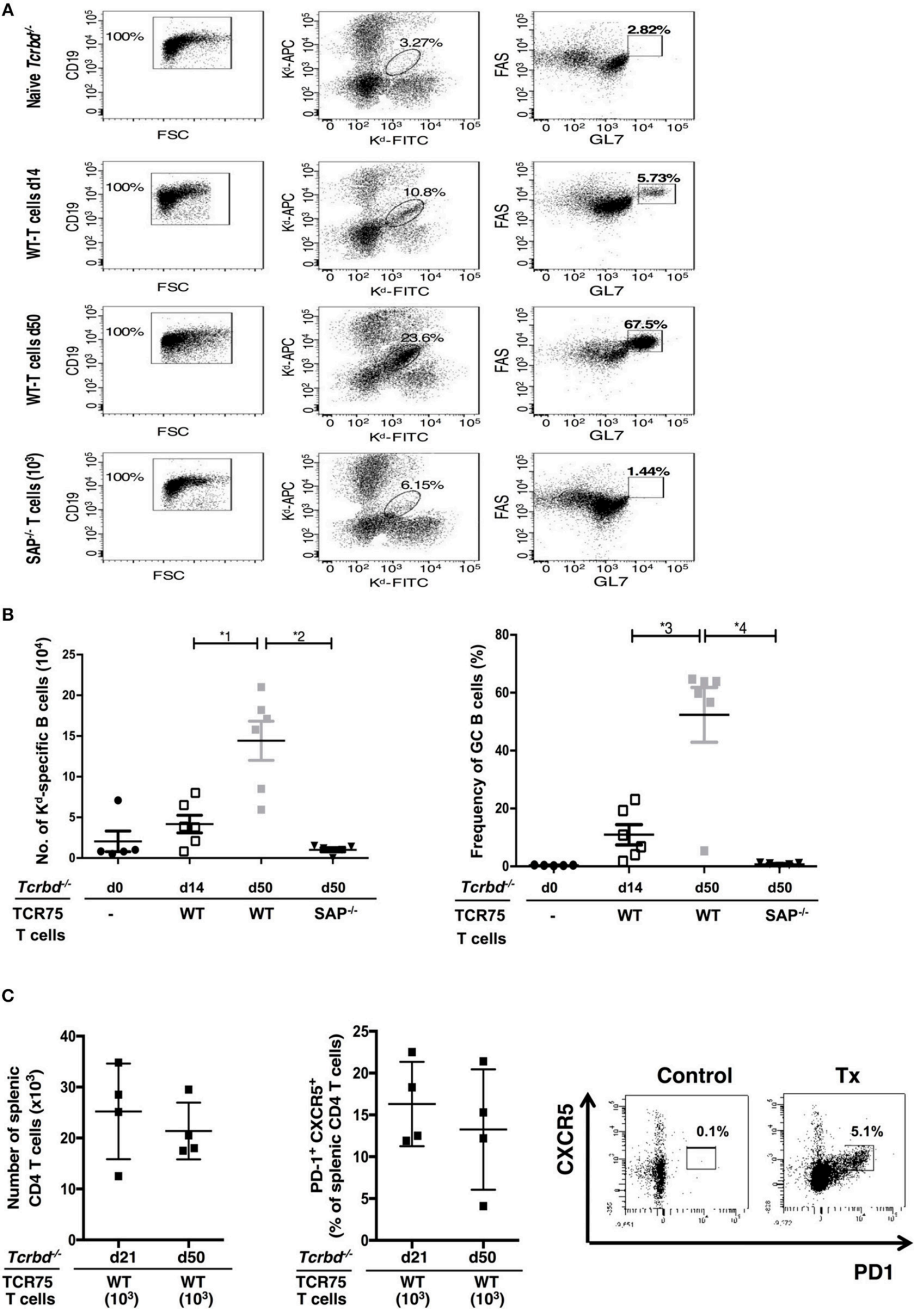
Characterization of allospecific B cells. **(A)**, Splenic H-2K^d^-specific B cells were identified by flow cytometric detection for binding of CD19^+ve^ B cells to FITC-conjugated and APC-conjugated synthetic H-2K^d^ tetramers in: naïve (un-reconstituted) BL/6 *Tcrbd*^−/−^ mice (*n* = 5); BL/6 *Tcrbd*^−/−^ mice, reconstituted with 10^3^ WT TCR75 CD4 T cells, 14 (*n* = 6) and 50 days (*n* = 6) after challenge with BALB/c heart allografts; and at 50 days after transplantation with BALB/c hearts and transfer of 10^3^ *Sh2d1a*^−/−^ TCR75 CD4 T cells (SAP^−/−^; *n* = 5). Gated cells in middle column of representative dot plots show percentage of enriched CD19^+ve^ B cells binding H-2K^d^ tetramer; right column shows percentage of GC-specific tetramer bound CD19^+ve^ B cells. **(B)** Histogram (left)—absolute numbers of splenic H-2K^d^-specific B cells (x 10^4^ cells per mouse) enumerated; histogram (right)—percentage of H-2K^d^-specific B cells expressing FAS^hi^GL7^+ve^ GC phenotype. Data represents mean ± S.E.M, with each dot representing the biological replicate in a separate animal; ^*^^1^*P* = 0.01, ^*^^2^*P* = 0.004, ^*^^3^*P* = 0.02 ^*^^4^*P* = 0.004 Mann-Whitney test. **(C)** Total numbers of splenic CD4 T cells (left histogram) and proportion displaying PD1^+^ CXCR5^+^ T_FH_ cell phenotype (right histogram) as determined by flow cytometry analysis at indicated time points following transplantation of *Tcrbd*^−/−^ recipients with a BALB/c heart graft and reconstitution with 10^3^ WT TCR75 CD4 T cells. Representative flow cytometry plot of unchallenged, but adoptively transferred (with 5 × 10^5^ TCR75 CD4 T cells) BL/6 *Tcrbd*^−/−^ mouse (left) and transplanted WT reconstituted recipient (right).

The GC response was further analyzed by using synthetic MHC class I H-2K^d^ tetramers to label individual allospecific B cells in recipient mice, as detailed previously (47–49). Flow cytometric characterization of the labeled allospecific B cell population revealed that in recipient mice 7 weeks after transplantation, ~40% of the H-2K^d^-specific splenic B cells displayed a GC phenotype (Figures 4A,B). In comparison, in the same group 2 weeks after transplant, numbers of total, and GC H-2K^d^-specific B cells were much fewer (Figures 4A,B). This is in keeping with the limited deposition of anti-K^d^ specific LLPCs in the bone marrow at this stage (Figure 3C).

The TCR-transgenic TCR75 CD4 T cells undergoe only minimal lymphopenia-induced homeostatic proliferation (38, 44), but would be expected to undergo robust antigen-specific expansion upon encounter of target H-2K^d^ allopeptide in transplanted recipients (50), with presumably, acquisition of T_FH_ cell phenotype. In this respect, flow cytometric analysis of CD4 T cells after transplantation (Figure 4C) confirmed the presence of transferred TCR75 T cells in spleens of recipient BL/6 *Tcrbd*^−/−^ mice. Reliable assessment of T cell phenotype was not possible at early time points (days 5–7), reflecting the small number of cells viable immediately after transfer, but at later time points, a sub-population that had acquired CXCR5^+^ PD1^+^ T_FH_ cell signature-phenotype (51) was evident (Figure 4C), although the precise proportion of T_FH_ cells varied considerably between recipient animals. This T_FH_ cell population presumably corresponded to those CD4 T cells identified within the GCs on immunofluorescent staining (Figure 3A, right panel).

**Figure 5:**
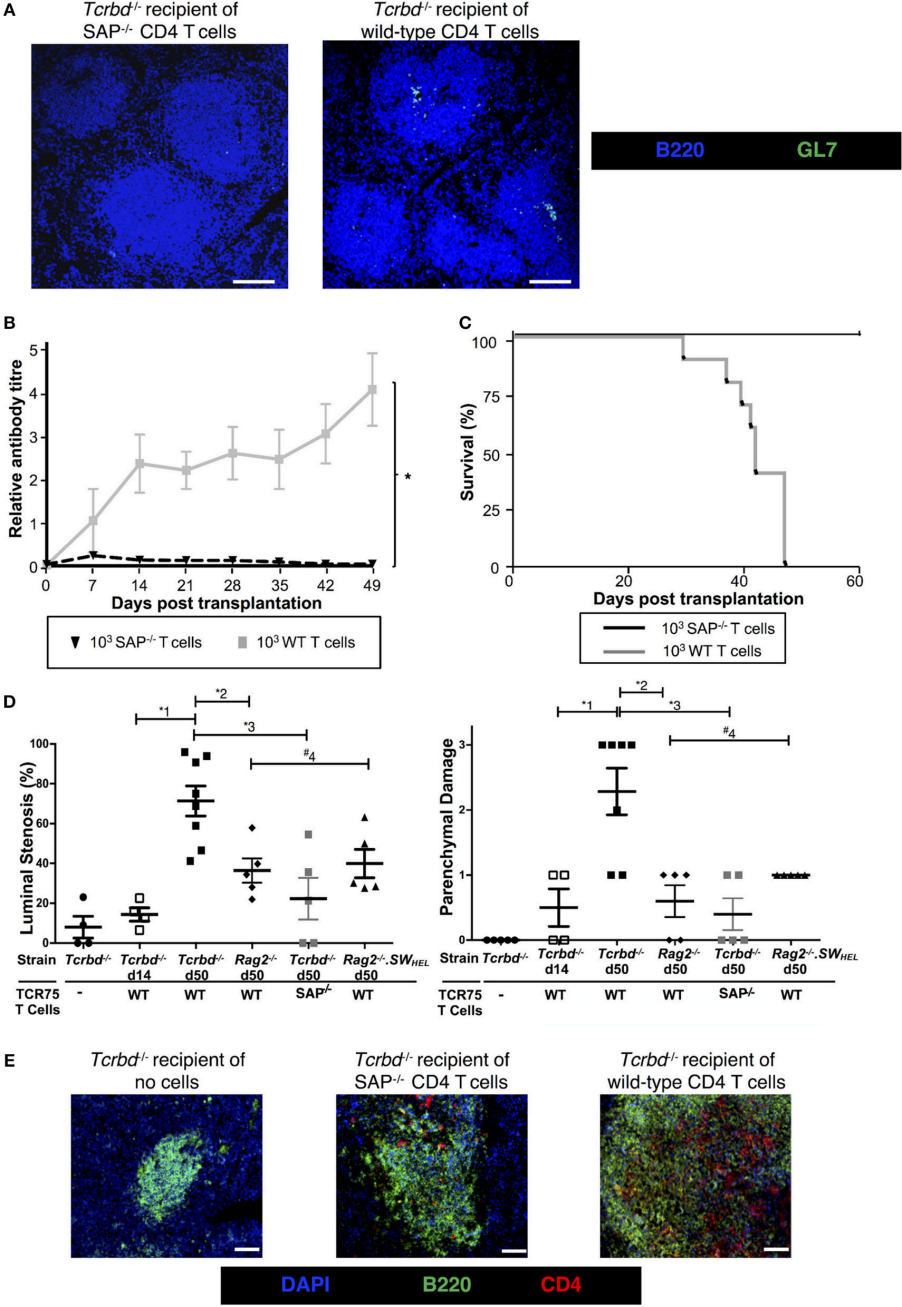
Germinal center alloantibody responses mediate chronic allograft rejection. **(A)** Representative low magnification confocal immunofluorescence photomicrographs of splenic B cell follicles stained with antibodies against B220 (B cells, blue) and GL7 (GC B cells, green) at d50 from BL/6 *Tcrbd*^−/−^ recipients of BALB/c heart grafts adoptively transferred with 10^3^ *Sh2d1a*^−/−^ (left) or wild-type (right) TCR75 CD4 T cells (scale bar – 200 μm). **(B)** Anti-H-2K^d^ alloantibody in recipients with 10^3^ SAP^−/−^ TCR75 CD4 T cells (*n* = 7) was markedly diminished compared to recipients given WT TCR75 CD4 T cells (*n* = 7); ^*^P < 0.001, two-way ANOVA. **(C)** Whereas BALB/c heart allografts survived long-term in BL/6 *Tcrbd*^−/−^ recipients reconstituted with 10^3^ SAP^−/−^ TCR75 CD4 T cells (*n* = 7), allografts were rejected in recipients given 1 × 10^3^ WT (SAP^+/+^) TCR75 CD4 T cells (*n* = 7, MST-50 days; *P* < 0.001, log-rank test). **(D)** Severity of vasculopathy (left) and parenchymal damage (right) in BALB/c hearts explanted: from unmodified (non-reconstituted) BL/6 *Tcrbd*^−/−^ mice (day 50, *n* = 4); at d14 (*n* = 4) and d50 (*n* = 8) from BL/6 *Tcrbd*^−/−^ recipients reconstituted with WT TCR75 CD4 T cells; at d50 from *Rag2*^−/−^ recipients reconstituted with WT TCR75 CD4 T cells (*n* = 5); at d50 from BL/6 *Tcrbd*^−/−^ recipients reconstituted with 10^3^ SAP^−/−^ TCR75 CD4 T cells (*n* = 5) and at day 50 from *Rag2*^−/−^ SWHEL recipients simultaneously transplanted with BALB/c heart and mHEL-Kd skin graft and reconstituted with 10^3^ TCR75 CD4 T cells (*n* = 5). Each dot represents a single animal; *Left*: ^*^^1^*P* = 0.001, ^*^^2^*P* = 0.008, ^*^^3^*P* = 0.003, ^#4^*P* = 0.29, *Right*: ^*^^1^*P* = 0.008, ^*^^2^*P* = 0.005, ^*^^3^*P* = 0.003, ^#4^*P* = 0.18; two-tailed Student's *t*-test for normally distributed and Mann-Whitney tests for non-parametric data. **(E)** Representative confocal immunofluorescence photomicrographs of splenic cryostat sections stained with antibodies against B220 (B cells, green) and CD4 (red) at day 50 after transplantation of BALB/c heart grafts into BL/6 *Tcrbd*^−/−^ recipients reconstituted with no cells (left), 10^3^ *Sh2d1a*^−/−^ (middle) or wild-type (right) TCR75 CD4 T cells (scale bar – 50 μm).

To confirm that GC activity was essential for mediating chronic rejection in this group, BL/6 *Tcrbd*^−/−^ recipients of BALB/c heart allografts were instead reconstituted with *Sh2d1a*^−/−^ TCR75 CD4 T cells the day after transplantation. Lacking the *Sh2d1a* gene, these T cells do not express SLAM-associated protein (SAP) and cannot undergo productive interaction with B cells for formation of T_FH_ cells (52). As expected, recipient BL/6 *Tcrbd*^−/−^ mice reconstituted with 10^3^ *Sh2d1a*^−/−^ TCR75 CD4 T cells did not develop splenic GC activity (Figure 3B, Figure 5A) and FAS^hi^GL7^+ve^ H-2K^d^-allospecific GC B cells were not detectable (Figures 4A,B). Compared to recipient mice reconstituted with similar numbers of wild-type (WT) TCR75 CD4 T cells, the anti-K^d^ alloantibody response in mice receiving *Sh2d1a*^−/−^ TCR75 T cells was markedly abbreviated (Figure 5B), with absence of LLPC deposition in the bone marrow (Figure 3C). Notably, unlike the chronic rejection observed in the WT group, BALB/c heart grafts survived long-term in the SAP^−/−^ recipients (Figure 5C), without evidence of endothelial complement deposition (Figure 2A) and with the development of only minimal allograft vasculopathy, and parenchymal injury (Figure 5D). The absence of GC activity was not due to early death of the transferred *Sh2d1a*^−/−^ TCR75 T cells, because they were detectable within the spleens of recipient BL/6 *Tcrbd*^−/−^ mice at late time points after transfer, albeit not as abundantly as following transfer of similar numbers of WT TCR75 CD4 T cells (Figure 5E). This possibly reflects continued proliferation relating to the robust GC response and ongoing graft injury in the recipient group reconstituted with WT TCR75 CD4 T cells (50).

### Distinguishing Alloantibody Production From Antigen Presentation in GC-Mediated Allograft Injury

**Figure 6:**
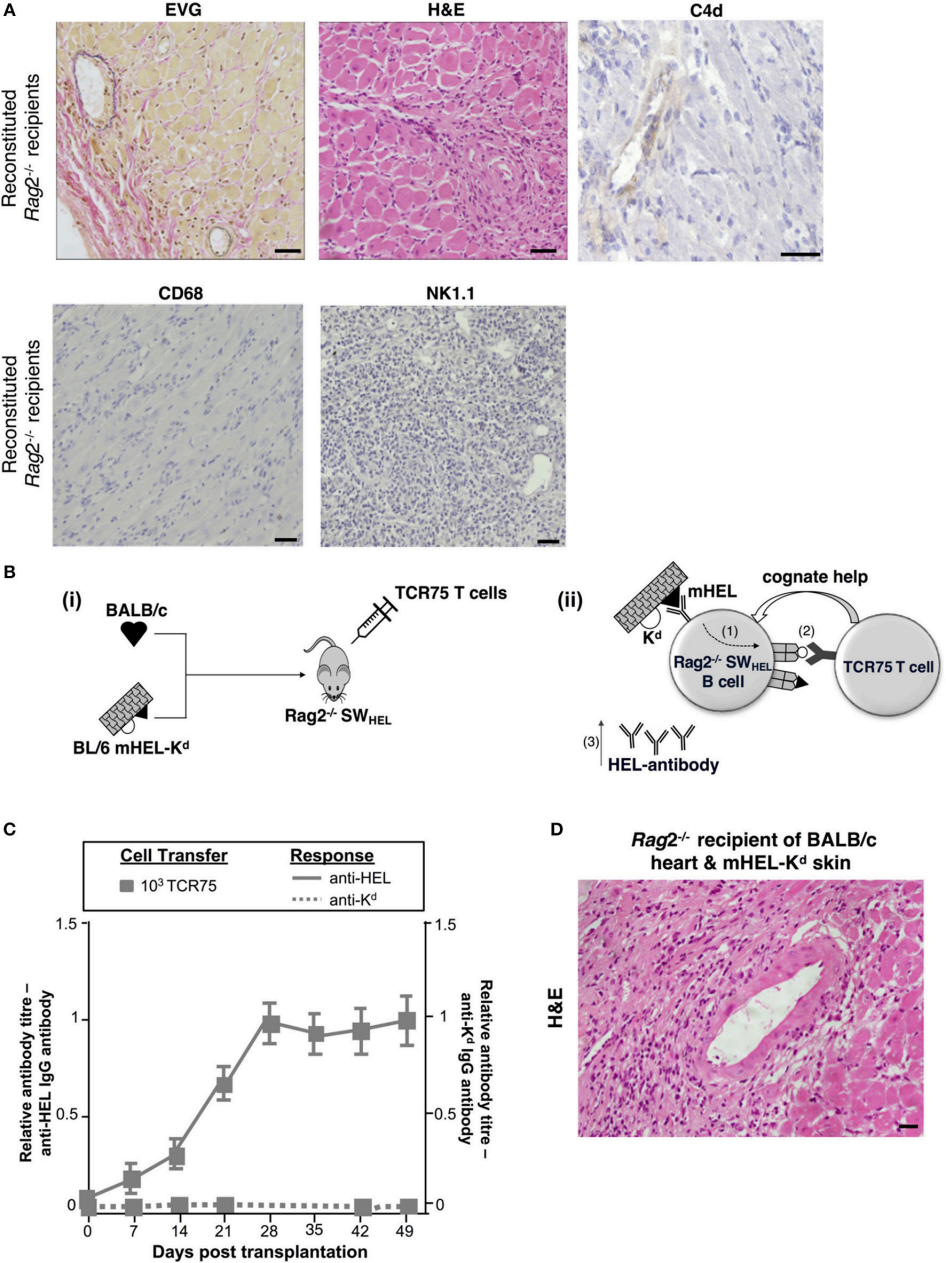
Contribution of alloantibody to allograft rejection. **(A)** Elastin van Gieson and H&E stained sections of d50 BALB/c heart allografts from reconstituted *Rag2*^−/−^ recipients with 10^3^ TCR75 CD4 T cells demonstrate focal areas of mild concentric vasculopathy and lymphocytic infiltrates *(EVG)* within well-preserved vasculature and myocardium (*H&E)*, with absence of capillary C4d staining and NK cell and macrophage infiltration. Scale bars: H&E, CD68, and NK1.1–50 μm; EVG−65 μm; C4d−100 μm. **(B)** Model to distinguish alloantibody production from alloantigen presentation. (i) BALB/c heart graft and BL/6 mHEL-K^d^ skin graft which co-express membrane-bound Hen Egg Lysosyme (HEL, black triangle) and MHC class I H-2K^d^ (white semicircle) were engrafted to a *Rag2*^−/−^ SW_HEL_ recipient, simultaneously reconstituted with TCR75 CD4 T cells. Critically, on the *Rag2*^−/−^ background, VDJ recombination is not possible and all B cells express a single HEL-specific BCR. (ii) Following challenge with a BL/6 mHEL-K^d^ skin graft, SW_HEL_ B cell recognition of mHEL target antigen on the skin graft is expected to result in additional internalization and processing of the H-2K^d^ alloantigen co-expressed on the surface of donor cells (step 1). CD4 T cell help is limited to the adoptively-transferred TCR75 CD4 T cells that recognize processed H-2K^d^ allopeptide. Hence, generation of an Ig-switched anti-HEL antibody response is dependent upon presentation of co-internalized H-2K^d^ antigen by the SW_HEL_ B cell for receipt of cognate help from the TCR75 CD4 T cell **(step 2)**. This results in generation of class-switched anti-HEL alloantibody **(step 3)**. **(C)** *Rag2*^−/−^SW_HEL_ recipients reconstituted with 10^3^ TCR75 CD4 T cells and simultaneously transplanted with a BALB/c heart and mHEL-K^d^ skin graft developed strong anti-HEL IgG (continuous lines), and undetectable anti-H-2K^d^ IgG (dotted lines). Data represent mean and S.E.M of n = 6 mice/group. [**(D)** Representative photomicrographs of H&E stained sections of d50 heart allografts [in **(C)**] depicting only mild vasculopathy and parenchymal injury (scale bar: 50 μm)].

The humoral features in explanted heart allografts, coupled to the absence of overt lymphocytic infiltrates, suggest that the role of GC humoral alloimmunity in mediating chronic rejection relates to production of effector alloantibody. However, a number of additional experiments were performed to confirm the contribution of B cells to rejection. Firstly, T and B cell deficient *Rag2*^−/−^ C57BL/6 mice were reconstituted with 10^3^ TCR75 CD4 T cells following transplantation with a BALB/c heart allograft. Heart allografts transplanted into the reconstituted *Rag2*^−/−^ recipients [which, as expected, did not mount an alloantibody response (not shown)] continued to beat until explant, and when compared to the reconstituted *Tcrbd*^−/−^ recipient group recipients, developed less severe vasculopathy and less extensive parenchymal damage (Figures 5D, 6A). Endothelial C4d deposition was not detectable in explanted heart allografts, nor was macrophage or NK cell infiltration (Figures 6A, 2D), suggesting these features are a direct consequence of alloantibody production. Nevertheless, BALB/c heart allografts in the reconstituted *Rag2*^−/−^ recipients displayed some features of chronic damage, with sparse intravascular lymphocytic infiltrates in medium and large epicardial arteries and diffuse myocyte loss and replacement fibrosis evident at late time points (Figures 5D, 6A). These features presumably represent low-grade cellular rejection.

The requirement for B cells in mediating chronic heart allograft rejection could conceivably reflect a principal role for B cells as antigen presenters for allospecific T cell activation, rather than as producers of alloantibody. To distinguish alloantibody production from alloantigen presentation, we devised a system whereby the B cell antibody output would not recognize determinants expressed on a heart graft, but nevertheless, the B cells would receive cognate help and activation from indirect-pathway CD4 T cells that recognize processed alloantigen from that heart graft (Figure 6B). BL/6 *Rag2*^−/−^ SW_HEL_ recipients, which lack all T cells, but contain a monoclonal population of B cells specific for HEL protein (36), were reconstituted with limiting numbers (10^3^) of TCR75 CD4 T cells and challenged simultaneously with a BALB/c heart graft and a BL/6 mHEL-K^d^ skin graft (that co-expresses membrane-bound HEL protein and MHC class I K^d^ antigen). We have shown previously that allospecific B cells can present allopeptide derived from graft alloantigens that are distinct from their target alloantigen, most likely because the additional alloantigen is internalized bound to target antigen via the B cell receptor (38). Hence, as indicated by the strong anti-HEL IgG antibody responses generated to the mHEL-K^d^ skin graft (Figure 6C), we reasoned that the SW_HEL_ B cells presented H-2K^d^ allopeptide both for activation of, and receipt of help from, the adoptively-transferred TCR75 CD4 T cells. This was associated with rapid rejection of the mHEL-K^d^ skin graft (MST 16 days). As control, without T cell reconstitution, anti-HEL antibody was not generated in the BL/6 *Rag2*^−/−^ SW_HEL_ recipients and skin and heart grafts survived indefinitely (not shown). Of note, no anti-K^d^ alloantibody was detected in the reconstituted BL/6 *Rag2*^−/−^ SW_HEL_ recipients (Figure 6C) and the simultaneously-transplanted BALB/c heart allografts survived until day 100, with development of mild vasculopathy and parenchymal injury, comparable in severity to that observed in similarly-reconstituted *Rag2*^−/−^ recipients (Figures 5D, 6D). These experiments thus provide further evidence that chronic AMR is mediated by a GC reaction, and principally through production of alloantibody.

### Alloantibody as an Effector of Graft Damage

**Figure 7:**
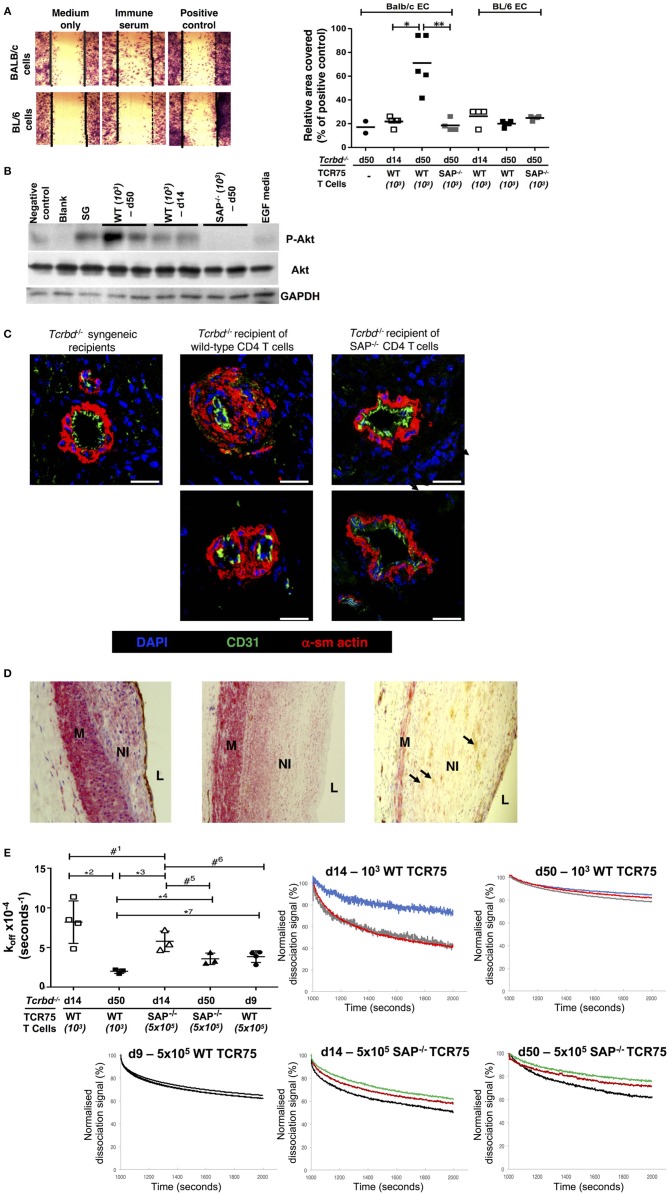
Alloantibody mediates endothelial activation and proliferative vasculopathy. (**A)** Representative photomicrograph of scratch-wound assay (left) and histogram (right) demonstrating enhanced migration of BALB/c endothelial cells (ECs) upon incubation with day 50 serum sampled from BL/6 *Tcrbd*^−/−^ reconstituted with wild-type (WT) or SAP^−/−^ TCR75 CD4 T cells, whereas BL/6 EC migration was not above background (mean values, with each dot representing an average of six high power fields analyzed per biological replicate). Migration of BALB/c ECs in response to sera from d14 WT or d50 SAP^−/−^ was significantly weaker compared to serum from d50 WT recipients; ^*^P = 0.01 and ^**^P = 0.02, Mann–Whitney tests. **(B)** Quiescent BALB/c endothelial cells stimulated with media containing epidermal growth factor (EGF) or column-purified sera from: d50 non-reconstituted BL/6 *Tcrbd*^−/−^ recipients of BALB/c grafts (negative control), d14 WT, d50 WT, d50 SAP^−/−^ recipients, and pooled hyperimmune anti-H-2K^d^ IgG serum (BALB/c skin grafts [SG] to BL/6 mice, positive control). Cell lysates were separated and immunoblotted with anti-Akt ^Ser473^, anti-phospho-Akt ^Ser473^, and GAPDH (control) mAb. Displayed Western blot represents three independent experiments. **(C)** Representative photomicrographs of coronary arteries from allografts explanted at day 50 and double immunolabeled with endothelial marker CD31 (green) and α-smooth muscle actin (α-sm actin, red); scale bar−200 μm. Increased neointimal lesions in chronically rejecting BALB/c allografts containing a large number of α-actin–positive smooth muscle cells were observed in BL/6 *Tcrbd*^−/−^ recipients reconstituted with 10^3^ TCR75 T cells (*middle column*) but not with SAP^−/−^ TCR75 CD4 T cells (right); *Tcrbd*^−/−^ syngeneic grafts shown as comparison (left). **(D)** Representative photomicrographs of endomyocardial biopsies of human heart allografts in recipients with antibody-mediated rejection and double-immunostained with the endothelial marker CD31 (brown) and α-smooth muscle actin (red); magnification x25. Pronounced neointimal (NI) lesions, composed of dense smooth muscle cells (pink), lying between the media (M) and arterial lumen (L). Remnants of endothelial cell layer seen within neointima (arrows, right image). Images representative of staining patterns observed in three individual patients. **(E)** Ranking anti-H-2K^d^ alloantibody dissociation kinetics. Left: dot plot histogram comparing anti-H-2K^d^ alloantibody dissociation constants (*k*_off_) among BL/6 *Tcrbd*^−/−^ recipients of BALB/c hearts reconstituted with either WT or SAP^−/−^ TCR75 CD4 T cells (at d9, 14, and 50 as depicted). Each value (mean ± SEM) represents the *k*_off_ after global fit of two serum dilutions of the same biological replicate (*n* = 3); ^#1^*P* = 0.21, ^*^^2^*P* = 0.01, ^*^^3^*P* = 0.008, ^*^^4^*P* = 0.02, ^#5^*P* = 0.06, ^#6^*P* = 0.05, ^#7^*P* = 0.01; two-tailed Student's *t*-test. *Right*: graphs depict sensograms of anti-H-2K^d^ antibody dissociation rates in sera from BL/6 *Tcrbd*^−/−^ recipients of BALB/c hearts reconstituted as shown, with each curve representing individual recipient mice.

A direct effector role for alloantibody on the allograft was then examined by testing the ability of sera from chronically-rejecting animals to activate cultured allogeneic endothelium, using an *in vitro* endothelial cell migration assay (45). Whereas test serum pooled from chronically-rejecting recipients sampled 7 weeks after transplant did not alter motility of syngeneic BL/6 endothelial cells, its addition to cultured BALB/c endothelial cells provoked migration similar to that observed following addition of positive control anti-H-2K^d^ IgG hyperimmune serum (Figure 7A). In contrast, only minimal BALB/c endothelial cell migration was observed upon addition of sera sampled from the same recipients at 2 weeks after transplantation (Figure 7A), even though a moderately strong anti-K^d^ IgG alloantibody response had developed by this time (Figure 1B). The endothelial cell migration observed possibly relates to signaling via the phosphoinositide 3-kinase (PI3K)/Akt pathway; a pro-survival and proliferative signaling cascade reportedly triggered by alloantibody binding to endothelial MHC class I (46, 53). Western blot analysis of the expression of phosphorylated Akt Ser^473^ in cultured BALB/c endothelial cells upon addition of test sera revealed similar signaling patterns; with strong Akt signaling observed upon addition of week 7 sera from chronically-rejecting recipients, but little or no signaling observed upon addition of either week 2 sera from the same recipients, or week 7 sera from the *Sh2d1a*^−/−^ reconstituted recipients (Figure 7B).

Although the mechanisms by which alloantibody provoke development of arterial disease in clinical solid-organ transplantation remain unclear (54), the allograft vasculopathy described is generally considered a disorder of neointimal proliferation. The *in vitro* endothelial responses observed in our model may therefore mimic the dysregulated endothelial activation and proliferative responses that are triggered by alloantibody binding in human transplant recipients (45, 46, 55–57). In this regard, staining for α-actin–positive smooth muscle cells revealed marked expansion of the smooth muscle cell component of the cardiac allograft microvasculature within the arterial neointima in hearts from chronically-rejecting recipients reconstituted with WT TCR75 CD4 T cells, but not in hearts transplanted into recipients adoptively transferred with *Sh2d1a*^−/−^ TCR75 CD4 T cells. (Figure 7C). Thus, GC humoral alloimmunity is associated with intra-allograft smooth muscle cell accumulation and proliferation resulting in intimal expansion. Of note, a similar staining pattern was present in heart allografts from human patients with a diagnosis of AMR (Figure 7D).

### The Germinal Center as a Source of Somatically-Mutated, High-Affinity Alloantibody

The above experiments detailing markedly augmented endothelial cell responses upon addition of sera sampled at late time points after rejection, even though seemingly robust titres of alloantibody were present at earlier time-points, suggests that the “quality” of the alloantibody response had possibly been improved by somatic hypermutation and affinity maturation. To examine this further, bio-layer interferometry (BLI) was performed, to rank H-2K^d^-specific antibody in the test sera according to binding strength (dissociation rate ranking). Because the precise concentration of H-2K^d^-specific antibody in the sera was not known, a *k*_off_ ranking method was employed (the kinetic rate of dissociation is concentration independent), with serum as the analyte and synthetic H-2K^d^ antigen immobilized on the sensor (58). As can be seen from Figure 7E (and see also Figure S1), test sera obtained from the week 7 chronically-rejecting group bound more strongly to target H-2K^d^ alloantigen (significantly lower dissociation constant *k*_off_) than sera obtained from the same animals at week 2, with less variability between individual recipient mice at the late time point. This decrease in the kinetic rate of dissociation observed over time is in keeping with GC-mediated affinity maturation of (allo)antibody responses (59). The minimal levels of alloantibody present at late time points in the recipients reconstituted with 10^3^ *Sh2d1a*^−/−^ TCR75 T cells prevented a similar BLI analysis, but day 14 sera from recipients reconstituted with 5 × 10^5^ *Sh2d1a*^−/−^ TCR75 T cells [that generated very robust, early extrafollicular responses (4)] bound with similar affinity to target H-2K^d^ antigen as sera sampled at the same time point from recipients reconstituted with 10^3^ WT TCR75 CD4 T cells (Figure 7E).

## Discussion

Although the durable nature of the alloantibody responses frequently observed in clinical transplantation is suggestive of an underlying GC response, this has not been demonstrated, largely because of difficulties in sampling the appropriate recipient tissue. The use of a murine model allowed us to demonstrate a critical role for the allospecific GC in the progression of allograft vasculopathy. Given the increasing emphasis on the contribution of alloantibody to chronic graft rejection, our results suggest that strategies that specifically target the GC humoral alloimmune response may improve long-term outcomes.

The critical role of the GC in progression of allograft vasculopathy in our model most likely reflects its role in producing long-lasting alloantibody responses (60), and it is notable that the LLPC subset (that can potentially produce antibody for the life of the individual) was evident in the bone marrow of the SAP-replete group, but not in the SAP-deficient group. Similarly, in a related piece of work using a different model of chronic rejection, we show the contribution of GC-mediated humoral autoimmunity to allograft vasculopathy independently of conventional alloimmunity, through a mechanism by which donor T cells initiate auto-reactive GC reactions that are then maintained by host T_FH_ cells (61, 62). However, it is possible that at least part of the GC contribution is ultimately T cell mediated, reflecting augmented allopeptide presentation and indirect-pathway CD4 T cell activation secondary to long-lived alloantibody production. Our experiments were not designed to exclude this possibility, and our findings using *Rag2*^−/−^ recipients highlight that cellular mechanisms can autonomously effect a degree of allograft vasculopathy and parenchymal damage. This would accord with Zeng et al. (63), who recently reported the contribution of B cells, independent of alloantibody production, in promoting allograft rejection by acting as antigen presenting cells for augmenting T cell activation. Notwithstanding, a number of our experiments support a direct effector role for alloantibody in the development of late parenchymal injury and allograft vasculopathy: allograft histology revealing clear evidence of humoral, but a lack of overt cellular, rejection; the association of alloantibody production and endothelial complement deposition; Akt signaling induced in allogeneic endothelial cells by exposure to sera from transplanted mice; and restoration of rejection by passive transfer of alloantibody into T and B cell deficient *Rag2*^−/−^ recipients [see also (4)]. Furthermore, it is notable that in our experiments involving recipients harboring a monoclonal population of B cells, in which a long-lasting antibody response was generated against irrelevant antigen, but in which B cell presentation to alloreactive CD4 T cells was required for its development, graft rejection was not observed, and the severity of allograft damage mirrored that observed in *Rag2*^−/−^ recipients. Our findings therefore accord with Hancock et al. (64) who described the development of murine cardiac allograft vasculopathy upon passive transfer of commercial anti-MHC class I alloantibody to T and B cell deficient recipients. We should, however, stress that the mechanisms by which the binding of alloantibody to allograft endothelium results in the neointimal proliferation and medial smooth muscle accumulations that typify allograft vasculopathy remain unclear, and will be the subject of ongoing investigation.

Labeling with synthetic MHC class I tetramer confirmed that transfer of SAP-replete helper CD4 T cells was associated with marked expansion in numbers of alloantigen-specific B cells, with approximately half of these cells expressing a GC phenotype. It is perhaps surprising that robust splenic GC activity was found at late time points after transplantation, in that GC responses against model protein antigen typically involute after about 3 weeks. Their continued presence implies ongoing delivery of help from T_FH_ cells; in support, confocal imaging confirmed CD4 T cells within secondary follicles at these late time points. The T_FH_ population differs from typical Th1 / Th2 subsets in that T_FH_ cells are relatively long-lived and their division limited, but their presence within the GC at 2 months from transplant presumably reflects ongoing differentiation from naïve precursors in response to the continued presence of target alloantigen (50). To what extent these late GC responses contribute to graft rejection is not clear; our experiments were principally designed to distinguish the GC from the extrafollicular response. As discussed above, the deposition of LLPCs in the bone marrow, one of the cardinal characteristics of the GC response, is likely a major factor in the progression of graft injury in our model. Such LLPC deposition would be expected to occur early in the GC response and it is conceivable that even a short-lived GC reaction would lead to eventual graft failure from the durable alloantibody production that ensues. Against this, as demonstrated in our model, as the GC response progresses, SHM produces alloantibody of increasing affinity for target alloantigen. This affinity maturation may be required for binding to target alloantigen on allograft endothelium with sufficiently strong affinity to trigger the proliferative response that is thought to underpin progression of allograft vasculopathy. In support, test sera sampled at late time points after transplantation in the help-limited group provoked pronounced Akt signaling. Hence, our findings suggest that late GC responses are necessary for progression of allograft vasculopathy, most likely reflecting the continual deposition of plasma cells that secrete alloantibody with increasing affinity for target alloantigen. Plasma cells that are produced from the GC at late time points would be expected to out-compete those already established in the bone marrow niche from earlier in the response (22), but it is also possible that they contribute to progressive allograft injury as short-lived cells.

Although the models developed for this study could be criticized for being artificially refined, we felt a reductionist approach, in which additional and potentially confounding effector mechanisms were excluded, was necessary to enable a definitive assessment of the role of the GC in chronic rejection. As such, this has allowed us to conclusively demonstrate the pivotal role of the T_FH_ cell subset in mediating progression of allograft vasculopathy by providing help for GC humoral immunity as well as confirming that the histological manifestations in the heart allografts that resemble those seen in clinical transplantation were a direct consequence of the alloantibody response. Our findings may therefore help to cement definitions of chronic AMR for clinical transplantation. The use of a monoclonal CD4 T cell population may, nevertheless, nullify any potential impact of either conventional regulatory, or follicular regulatory, T cells that would otherwise be present within wild-type, polyclonal populations. Against that, we have previously shown that only a small proportion of transferred TCR75 CD4 T cells adopt a T_FH_ phenotype (44) and our ongoing work has confirmed that other helper T cell subsets, including *FoxP3*-expressing regulatory CD4 T cells, differentiate within the transferred TCR75 T cell population. Likewise, Aloulou et al have recently described the development of T follicular regulatory cells within a monoclonal population of TCR-transgenic CD4 T cells (65). Our findings will therefore likely apply to wild-type recipients that harbor a polyclonal T cell repertoire. In support, we have recently reported that chronic rejection of “bm12.K^d^.IE” heart grafts is associated with late splenic GC responses in wild-type C57BL/6 recipients (50, 66). Similarly, our ongoing investigations have confirmed that GC activity is present at late time points in C57BL/6 recipients of chronically-rejecting MHC class II-mismatched “bm12” heart allografts and in C57BL/6 recipients of BALB/c heart grafts in which acute rejection is prevented by blocking co-stimulation signaling at the time of transplant. Possibly, the chronic alloresponse favors polarization to a T_FH_ cell phenotype, as has been reported for exhausted CD4 T cells responding to chronic viral infection (67). This perhaps explains the late development of alloantibody that is observed in human transplant patients.

We believe there are several aspects to our findings that have implications for clinical transplantation. Most obviously, our description of the role of GC humoral alloimmunity in the progression of allograft vasculopathy appears to mirror the development of durable donor specific alloantibody responses late (months or years) after solid-organ transplantation, and which are increasingly associated with poor graft outcomes (11, 68). Our results suggest these responses are likely to be outputs of the GC response, and further highlight the potential for developing strategies that specifically target the T_FH_ subset as a means of preventing late alloantibody-mediated allograft rejection. Because the T_FH_ cell subset represents only a small proportion of the total CD4 T cell population, this approach offers the potential advantage of preserving general immunocompetence, and could possibly be achieved, for example, by transfer of either allopeptide-specific (69). or donor derived regulatory CD4 T cells (70).

## Data Availability

The raw data supporting the conclusions of this manuscript will be made available by the authors, without undue reservation, to any qualified researcher.

## Author Contributions

All authors contributed extensively to the work presented in this paper. MC and JA jointly conceived the study with RM and GP, designed and implemented the cardiac allograft rejection model with contributions from MQ, MM, JMA, IG, and ML contributed to characterization of germinal center responses, and VK analyzed the BLI data. EM and MG performed cardiac allograft histopathological characterization. GP and RM prepared the manuscript, with contributions from MC, JA, VK, and ML.

### Conflict of Interest Statement

The authors declare that the research was conducted in the absence of any commercial or financial relationships that could be construed as a potential conflict of interest.
