# Supplementary material for: Germinal Center Alloantibody Responses Mediate Progression of Chronic Allograft Injury
Source: Front Immunol. 2019 Jan 23;9:3038. doi: 10.3389/fimmu.2018.03038 (PMC6351502; doi:10.3389/fimmu.2018.03038)
Supplement: Supplementary file 1 [file Image_1.pdf]

SUPPLEMENTAL DATA

Figure S1

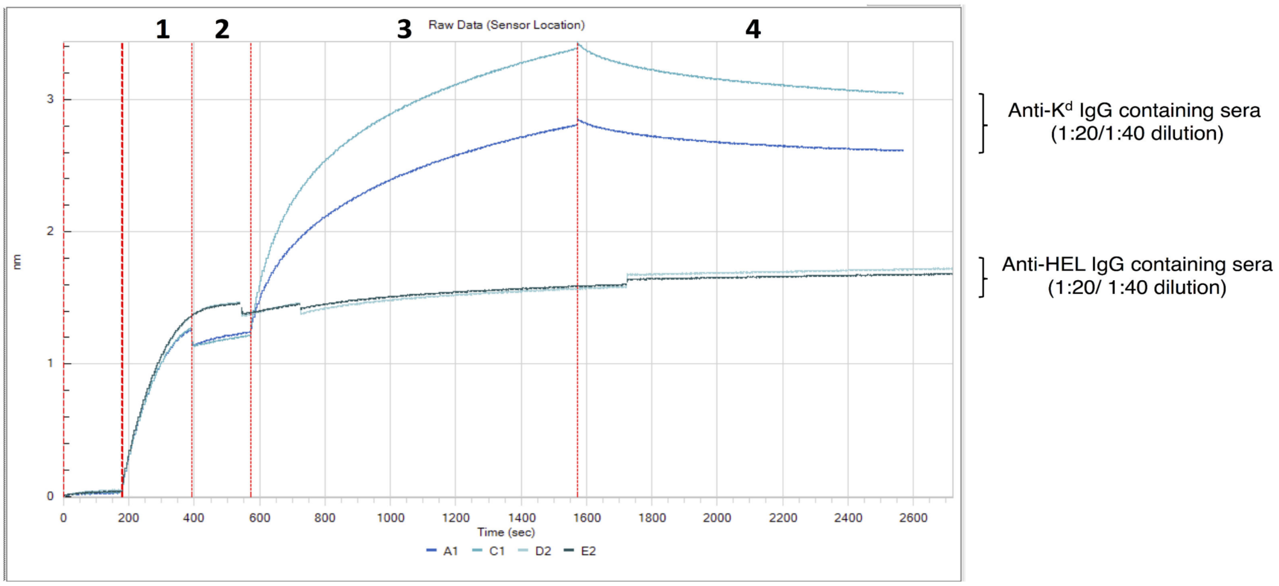

1: sensor loading with biotinylated MHC Class I H-2K^d
2: blocking in naïve serum (1:20/1:40)
3: association with test serum
4: dissociation in naïve serum (1:20/1:40)

Figure S1. Representative raw data binding kinetics of test sera containing H-2K^d alloantibody.

Streptavidin-coated sensors were moved between wells and binding kinetics were plotted in real time after baseline establishment and sensor pre-equilibration in the following order: **1)** loading with biotinylated H-2K^d protein, **2)** 2nd baseline establishment and blocking with naïve BL/6 *Tcrbd*$^{-/-}$ serum (diluted at 1:20 or 1:40), **3)** association with test serum diluted at 1:20 or 1:40 (shown here from day 50 wild-type help-limited *Tcrbd*$^{-/-}$ recipients [top two curves] or from BL/6 SW$_{HEL}$ recipients challenged with hearts from BL/6.mHEL mice [bottom two curves]), and **4)** dissociation in naïve serum at a dilution equivalent to test serum. Sonograms were corrected for inter-step alignment on Y-axis, and baseline drift was corrected by subtracting the average value obtained from reference sample (H-2K^d loaded sensor exposed to naïve B6 *Tcrbd*$^{-/-}$ serum) and reference sensor (unloaded sensor [no H-2K^d protein] exposed to naïve B6 *Tcrbd*$^{-/-}$ serum).
